# Association Between Temperature Management Using Passive Thermal Insulation and a Modified Aldrete Score During Cryoablation of Paroxysmal Atrial Fibrillation Under General Anesthesia

**DOI:** 10.1002/joa3.70460

**Published:** 2026-09-24

**Authors:** Arisa Yamaguchi, Masao Takemoto, Takuya Nagata, Reimi Goya, Yoshie Sadasue, Kaori Nagaoka, Norie Terauchi, Hijiri Inoue, Takahiro Mito, Michiko Kurachi, Takuya Tsuchihashi, Kyoko Matsuno, Tetsuji Okamoto

**Affiliations:** ^1^ Nursing Department Social Medical Corporation Steel Memorial Yawata Hospital Kitakyushu Japan; ^2^ School of Medical Sciences University of East Asia Shimonoseki Japan; ^3^ Cardiovascular Center Social Medical Corporation Steel Memorial Yawata Hospital Kitakyushu Japan; ^4^ Department of Cardiology Hakujuji Hospital Fukuoka Japan

**Keywords:** blanket, cryoablation, general anesthesia, hypothermia, modified Aldrete score, passive warming

## Abstract

**Background:**

Unintentional intraoperative hypothermia (core temperature < 36°C), a common and frequently preventable complication in patients undergoing surgery under general anesthesia (GA), involves adverse effects on coagulation and prolonged recovery from GA. Thus, this study aimed to clarify the effect of passive thermal insulation (PTI) with a heat insulation blanket (HIB) on successful early recovery from GA in patients undergoing cryoablation for atrial fibrillation (AF) under GA.

**Methods:**

Fifty‐five patients undergoing cryoablation under GA for nonvalvular paroxysmal AF were prospectively assigned to the Blanket (*n* = 28) or Control (conventional towelettes; *n* = 27) group. The esophageal temperature was measured using an esophageal temperature probe before and after cryoablation. The esophageal hypothermia was defined as a temperature < 36°C. The Δ in esophageal temperature was defined as the difference between the pre‐ and post‐anesthesia temperature. The primary endpoint of this study was successful early recovery from GA, defined as a Modified Aldrete score (MAS) ≥ 9 at 2 h after the discontinuation of sedation/anesthesia in patients undergoing cryoablation under GA.

**Results:**

This study demonstrated that the PTI using HIBs significantly reduced the incidence of esophageal hypothermia from 93% to 68% and increased the rate of successful early recovery from GA from 37% to 64%.

**Conclusions:**

PTI using HIBs was associated with a smaller decline in esophageal temperature and a higher proportion of patients achieving MAS ≥ 9 at 2 h after discontinuation of anesthesia during cryoablation under GA. Larger studies using validated core temperature measurements and clinically meaningful recovery endpoints are warranted.

**Trial Registration:**

This study was registered in the University Hospital Medical Information Network Clinical Trial Registry of Japan (UMIN000057904)

## Graphical abstract

This study demonstrated that the Δ in core temperature was significantly lower in the Blanket (blue bar) group than in the Control (red bar) group and the passive thermal insulation using heat insulation blankets significantly decreased the rate of hypothermia‐induced unsuccessful early recovery from general anesthesia from 64% to 37%. The numerical results are expressed as mean ± standard deviation.

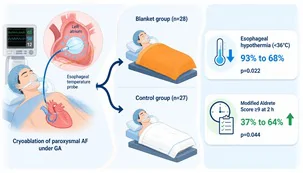

**Abbreviations**
ACT: activated clotting time
AF: atrial fibrillation
BIS: bispectral
CTI: cavo‐tricuspid isthmus
GA: general anesthesia
HIB: heat insulation blanket
LA: left atrium
LIPV: left inferior pulmonary vein
LSPV: left superior pulmonary vein
MAS: Modified Aldrete score
NOAC: non–vitamin K oral anticoagulants
PACU: post‐anesthesia care unit
PTI: passive thermal insulation
PV: pulmonary vein
PVAI: pulmonary vein antrum isolation
RIPV: right inferior pulmonary vein
RSPV: right superior pulmonary vein

## 1 Introduction

Unintentional intraoperative hypothermia (core temperature < 36°C), a common and frequently preventable complication in patients undergoing surgery under general anesthesia (GA), involves adverse effects on coagulation and drug metabolism as well as a prolonged recovery time from GA and discharge from the post‐anesthesia care unit (PACU) [1, 2, 3, 4]. Therefore, active and/or passive warming during the perioperative period is required to reduce the risk of perioperative hypothermia [1, 2, 3, 4]. Cryoablation [5, 6, 7, 8] is a useful treatment strategy for atrial fibrillation (AF) despite the current trend of decreasing balloon‐based ablation volumes due to pulsed‐field ablation [9]. Cryoablation involves freezing the cardiac tissue at −60°C to −70°C [5, 6, 7, 8]. Thus, physicians often observe a decrease in esophageal temperature to < 36°C measured by an esophageal temperature probe in patients despite completion of the cryoablation procedure. However, the prevalence and adverse effects of cryoablation‐related hypothermia during GA remain unknown. Thus, this single‐center randomized clinical trial aimed to clarify the effect of passive thermal insulation (PTI) using a heat insulation blanket (HIB) on successful early recovery from GA in patients undergoing cryoablation for AF under GA.

Concern was recently expressed about the safety and efficacy of using a Modified Aldrete score (MAS) [10] ≥ 9 (Supporting Information) to judge whether postoperative patients have recovered safely from GA and determine when they can be discharged from the PACU. Thus, the primary study endpoint was a successful early recovery from GA, which was defined herein as a MAS ≥ 9 at 2 h after the discontinuation of sedation/anesthesia. The secondary study endpoints were time to hemostasis and procedure‐associated complications.

## 2 Materials and Methods

### 2.1 Study Population and Laboratory Analysis

**FIGURE 1:**
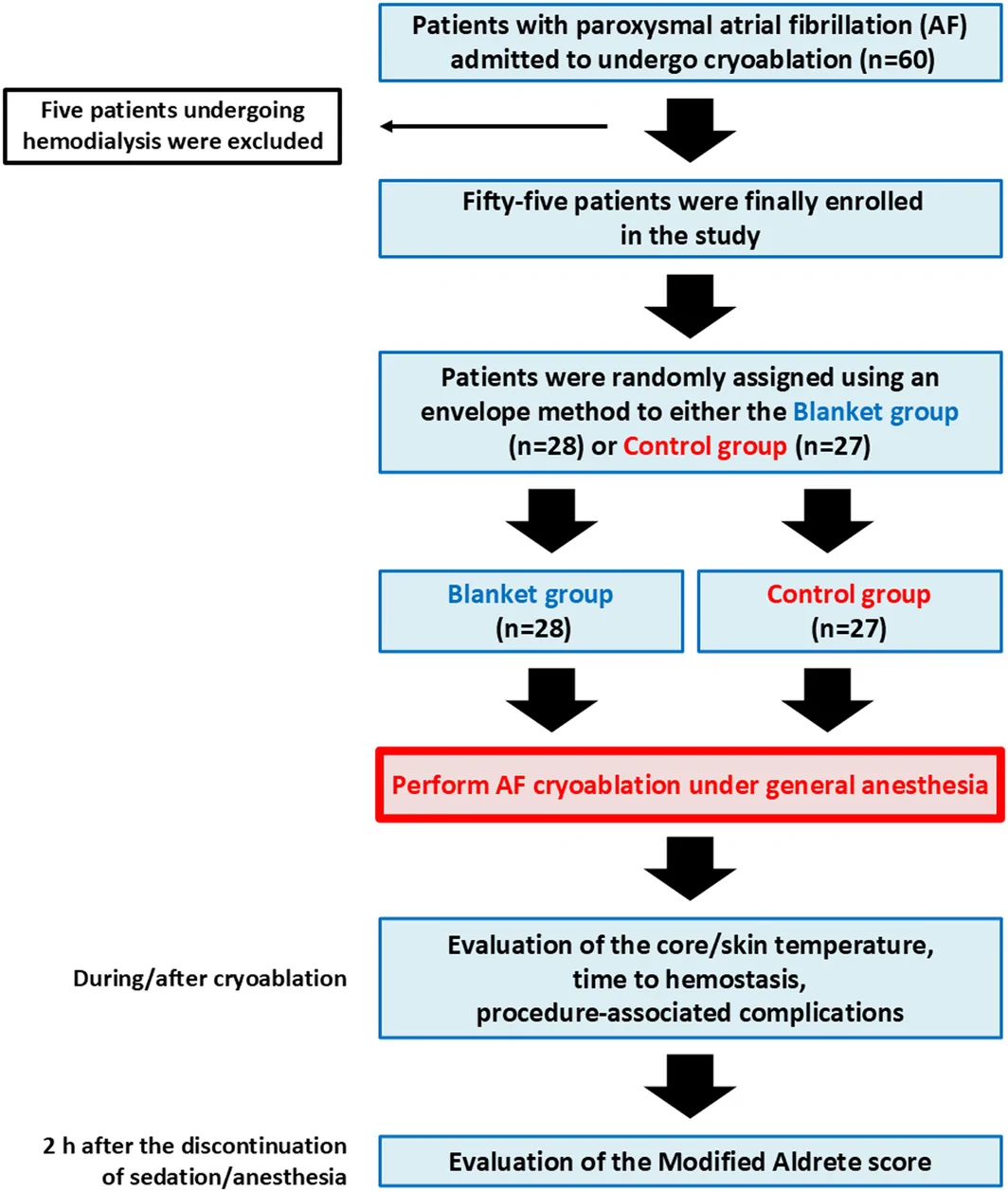
Study protocol.

From November 2022 to May 2024, 60 patients with nonvalvular paroxysmal AF were admitted to our hospital for AF cryoablation and were prospectively evaluated (Figure 1). Patients with a history of AF ablation, myocardial infarction, cardiac surgery, structural heart disease, or hemodialysis were excluded. As a result, five patients undergoing hemodialysis were excluded, and 55 patients were finally enrolled in the study. The AF type was determined according to the 2020 Japanese Circulation Society/Japanese Heart Rhythm Society guidelines for pharmacotherapy of cardiac arrhythmias [11]. Paroxysmal AF was defined as AF that occurred for a few minutes to days and stopped on its own within 7 days. All patients had their medical histories recorded and underwent a physical examination, laboratory analysis, chest radiography, 12‐lead electrocardiography, echocardiography, and CHADS_2_ score assessment [11, 12], within 1 month before admission.

### 2.2 Temperature Management Using HIBs

The 55 patients with paroxysmal AF were randomly assigned using the envelope method to undergo PTI using an HIB (Blanket group; *n* = 28) or conventional towels (Control group; *n* = 27) (Figure 1).

**FIGURE 2:**
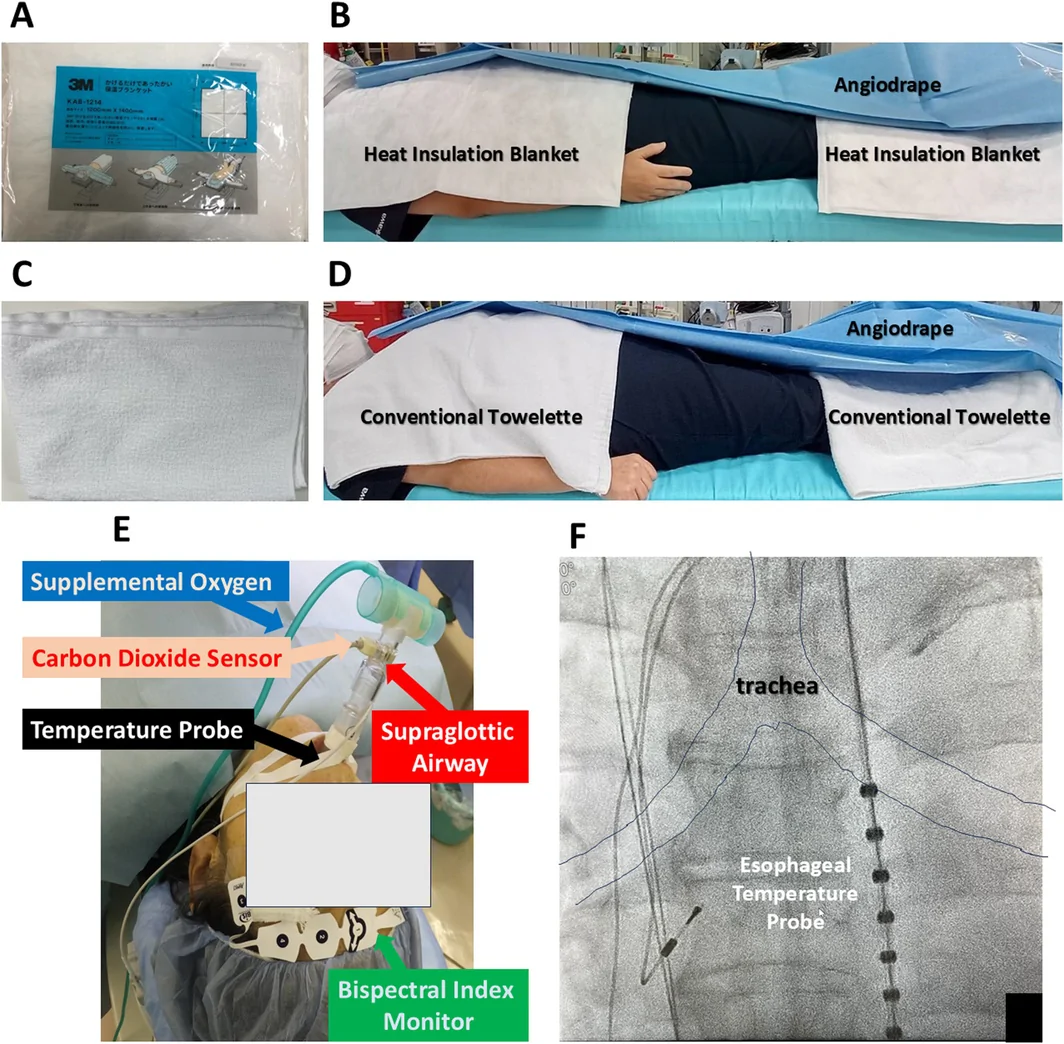
Heat insulation blanket (A, B) and conventional towelette (C, D). Photograph of a patient with a supraglottic airway (SGA; i‐gel) (red arrow) in whom general anesthesia was monitored using a bispectral index monitor (green arrow) placed on the forehead (E). Supplemental oxygen (blue arrow) was routinely used via the SGA. An exhaling carbon dioxide sensor (pink arrow) was attached to the SGA. A temperature probe was inserted through the nostril of the patient via a side hole of the SGA to monitor the esophageal temperature (black arrow). Fluoroscopic imaging of the chest (F).

Thinsulate (3 M, Tokyo, Japan) (Figure 2A), a newly developed HIB made of a high‐performance batting material, offers superior heat retention compared to conventional towels (Figure 2C) and measures 1200 mm × 1400 mm. The conventional towels were made of cotton and measured 680 mm × 1320 mm. Prior to GA induction, one HIB (Figure 2B) or conventional towel (Figure 2D) was placed over the trunk and the patient's legs were covered with a sterile drape (Angiodrape; Livedo Medical, Osaka, Japan).

### 2.3 Ablation Procedure

Informed consent was obtained from all patients. Each ablation was performed as previously described [13]. Briefly, the patients underwent GA, and a supraglottic airway (i‐gel; Intersurgical Ltd., Wokingham, Berkshire, UK) was inserted and managed with mechanical ventilation under the intravenous administration of propofol (bolus dose of 1 mg/kg and maintenance dose of 4 mg/kg to 10 mg/kg per hour) and dexmedetomidine (maintenance dose of 0.2 μg/kg to 0.7 μg/kg per hour). The bispectral index (BIS) was maintained at 40–60 using a BIS index monitoring system (A‐3100C; NIHON KODEN, Tokyo, Japan) with control of the oxygen saturation (SpO_2_) and end‐tidal carbon dioxide (EtCO_2_) at 96–100% and 35–45 mmHg, respectively, as previously described [13] (Figure 2E). The BIS and SpO_2_ and EtCO_2_ levels were recorded every 5 min. A 140 U/kg dose of heparin was administered upon venous access, and heparinized saline was infused to maintain an activated clotting time (ACT) of 300–400 s.

During the procedure, ACT was measured and recorded every 15 min. Femoral arterial access (3‐Fr sheath) was routinely performed to continuously monitor blood pressure (BP) and heart rate. When the BP decreased to < 100 mmHg, intravenous dopamine was initiated to maintain a BP of 100–130 mmHg. A 7‐Fr, 20‐pole, three‐site catheter (BeeAT; Japan LifeLine, Tokyo, Japan) was inserted through the left femoral vein for pacing, recording, and internal cardioversion. Using the left femoral venous access site, a single transseptal puncture was carefully performed using a radiofrequency needle (Baylis Medical Inc., Montreal, Canada) inserted through a 10‐Fr sheath via the left femoral vein under the guidance of intracardiac echocardiography (ViewFlex Xtra ICE catheter; Abbott, St. Paul, MN, USA). An 8.5‐Fr sheath (Abbott, St. Paul, MN, USA) was inserted through the right femoral vein, placed in the left atrium (LA), and subsequently replaced with a 15‐Fr steerable transseptal sheath (FlexCath Advance, Medtronic CryoCath LP, Minneapolis, MN, USA).

In all patients, a second‐generation 28‐mm cryoballoon catheter (Arctic Front Advance; Medtronic CryoCath LP) was used for pulmonary vein (PV) antrum isolation (PVAI). The cryoballoon was maneuvered to all PV ostia using a steerable 15‐Fr sheath and an Achieve catheter (Medtronic CryoCath LP) inserted through the lumen of the balloon catheter. A 6‐Fr 20‐pole catheter (Livewire; Abbott) was inserted through the left femoral vein, and the right PN was constantly paced using a cardiac stimulator system (SEC‐5104; NIHON KODEN, Tokyo, Japan). After the intravenous administration of atropine sulfate (0.5 mg), the balloon was inflated in the LA and directed toward the PV ostia. Balloon occlusion was assessed by injecting 50% diluted contrast through the central lumen of the cryoballoon catheter.

Cryoablation was continued for 180 or 240 s in the right inferior PV (RIPV), right superior PV (RSPV), left inferior PV (LIPV), and left superior PV (LSPV). The procedure began systematically with RIPV, followed by RSPV, LSPV, and LIPV. Successful PVAI was defined as a bidirectional (entrance and exit) block of all four pulmonary veins (PVs) as verified by mapping and pacing with the ring electrode of the Achieve catheter. If needed, additional touch‐up applications with an open irrigated 3.5‐mm‐tip ablation catheter (FlexAbility; Abbott) were delivered after cryoablation of all PVs. Furthermore, if common atrial flutter was previously documented or induced by programmed stimulation after cryoablation, additional cavotricuspid isthmus (CTI) ablation was performed using an open irrigated 3.5‐mm‐tip ablation catheter (FlexAbility; Abbott).

### 2.4 Evaluation of Eardrum and Esophageal Temperature During Cryoablation in the Preliminary Study (Figure 3)

**FIGURE 3:**
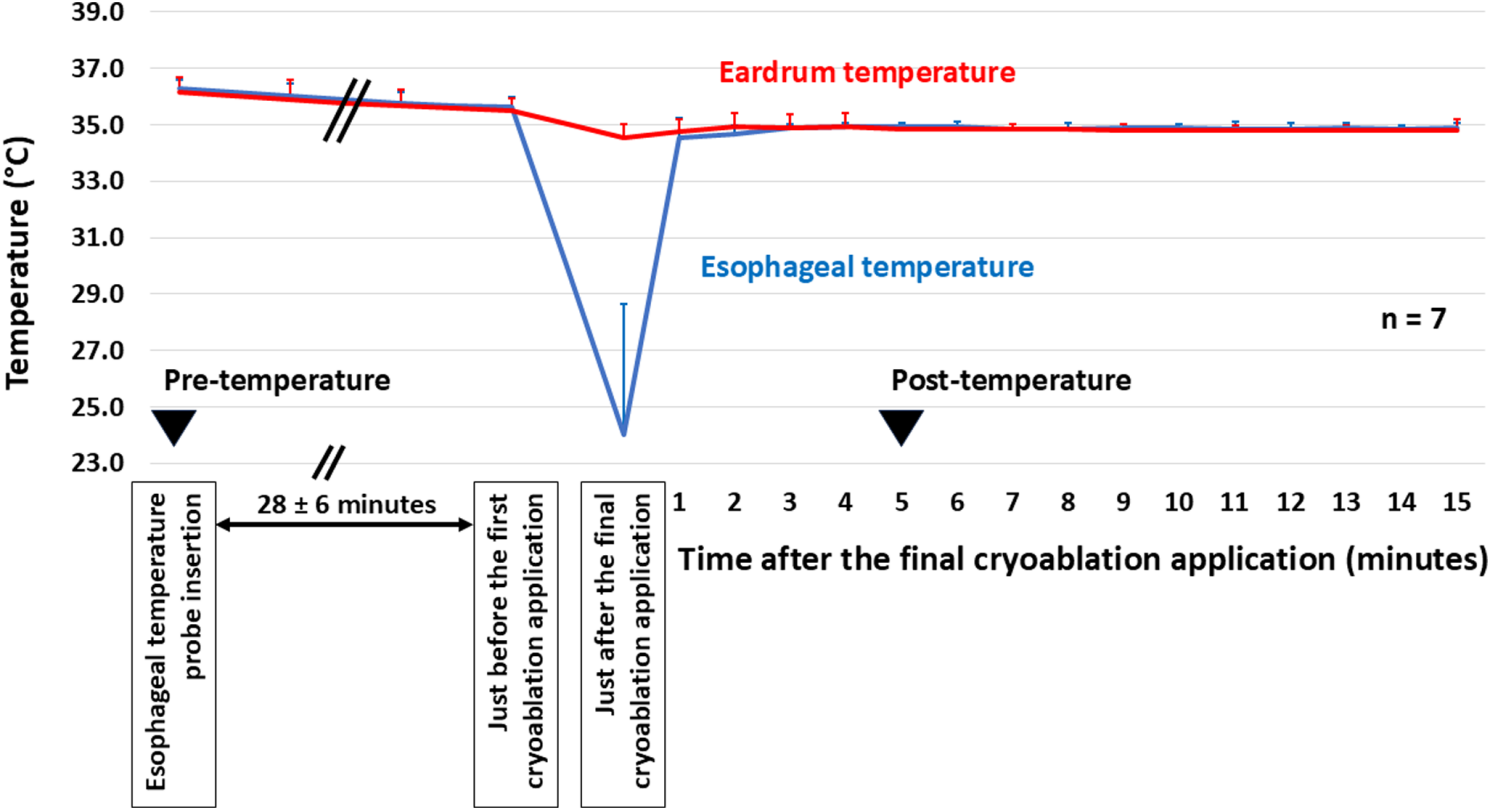
Time courses of the eardrum (red line) and esophageal (blue line) temperature after insertion of the esophageal temperature probe during cryoablation under general anesthesia in the preliminary study (*n* = 7).

We demonstrated that the time courses of the eardrum and esophageal temperatures after insertion of the esophageal temperature probe during cryoablation under GA in our preliminary study (Figure 3; *n* = 7). Under GA, the eardrum and esophageal temperatures gradually decreased after the insertion of the esophageal temperature probe. During cryoballoon application, the esophageal temperature markedly decreased compared to the eardrum temperature. After cryoballoon application, the esophageal temperature increased to the same level as the eardrum temperature within 3 min, and both the eardrum and esophageal temperatures plateaued at least 15 min after cryoballoon application. These findings indicate that the esophageal temperature may indicate the core temperature 3 min after the final cryoballoon application. Thus, the esophageal temperature measured 5 min after the final cryoablation application was defined as the ‘post‐esophageal temperature’.

### 2.5 Evaluation of Hypothermia

The room temperature was set at 24°C. Once the BIS had decreased to < 50, an esophageal temperature probe (SensiTherm; Abbott) was inserted through the side hole of the i‐gel and positioned between the left superior and inferior PVs to monitor the patient's esophageal temperature (Figure 2F). To avoid esophageal damage during the cryoablation, the energy supply was discontinued when the temperature fell to < 15°C. During the procedure, the patient's esophageal temperature was measured using an esophageal temperature probe, while skin temperature was measured at the axilla. Measurements were obtained immediately after probe insertion which was defined as the ‘pre‐temperature’ and 5 min after the final cryoablation application which was defined as the ‘post‐temperature’ (Figure 3). Esophageal or skin hypothermia was defined as a temperature < 36°C measured using an esophageal or axillary temperature probe. The Δ in esophageal or skin temperature was defined as the temperature decreases from the pre‐ to post‐temperatures (post‐ minus pre‐temperature). After the discontinuation of the intravenous administration of propofol, dexmedetomidine, and dopamine, the administered volumes were recorded.

### 2.6 Post‐Procedural Hemostasis and MAS Evaluation

After completing ablation and treatment with protamine sulfate, we removed both sheaths (15‐Fr FlexCath Advance; Medtronic CryoCath LP) from the right femoral vein and immediately applied constant manual compression using a newly developed hemostatic pad, Nepcell S (Alliance Medical Group Co. Ltd., Kanagawa, Japan), which accelerates the clotting cascade in the vessels [14]. Sequentially, we removed the sheaths from the left femoral vein (7‐Fr and 10‐Fr) and artery (3‐Fr) and immediately applied constant manual compression using a Nepcell S pad applied to the sites. The time to hemostasis, defined as the time from removal of the first sheath to complete hemostasis, was measured. Immediate hemostasis was achieved in the catheter laboratory, and a pressure bandage was applied using a gauze ball. After the procedure, the patients were transferred to the PACU. Two hours after the discontinuation of sedation/anesthesia in the PACU, the MAS and prevalence of dopamine discontinuation were evaluated. A MAS ≥ 9 was the single criterion for PACU discharge.

### 2.7 Statistical Analysis

The numerical results are expressed as mean ± standard deviation. Intergroup statistical analyses were performed using Fisher's exact test or repeated measures analysis of variance. Normality of the single‐time‐point continuous variables in Tables 1 and 2 was assessed separately within each study group using the Shapiro–Wilk test. Variables for which normality was not rejected in either group were summarized as mean ± standard deviation and compared using a two‐sided independent‐samples Student's *t*‐test. Variables for which normality was rejected in either group were summarized as median (interquartile range) and compared using a two‐sided Mann–Whitney *U* test. Analyses were based on available observations, and variable‐specific sample sizes were reported for variables with missing data. Pearson correlation coefficients between the Δ in esophageal or post‐esophageal temperature and total cooling time during cryoablation were determined using a simple linear regression analysis. The association between study group and achievement of a MAS ≥ 9 at 2 h after discontinuation of sedation/anesthesia was evaluated using logistic regression (Table 3). Model 1 included the study group alone and represented primary unadjusted comparisons. Model 2 was a post hoc sensitivity analysis adjusted for the baseline variables of age and left ventricular ejection fraction (LVEF), which were selected on clinical grounds without reference to univariable *p* values. Model 3 further adjusted for cumulative intra‐procedural propofol exposure (mg/kg) and dexmedetomidine exposure (μg/kg). Because anesthetic exposure was measured after randomization and may be on the causal pathway, Model 3 was considered a post hoc exploratory conditional sensitivity analysis and was not interpreted as an estimate of the total effect of the blanket assignment. Post‐core temperature measurements were not included as adjustment variables in the treatment‐group comparison models. Odds ratios (ORs), 95% confidence intervals (CIs), and two‐sided Wald *p* values were reported. Exploratory single‐marker receiver operating characteristic (ROC) analyses were performed for Δ in esophageal temperature, post‐esophageal temperature, and LVEF in relation to the achievement of MAS ≥ 9. The apparent areas under the curve (AUCs) and percentile 95% CIs were estimated using 5000 outcome‐stratified bootstrap resamples. Bootstrap optimism was estimated for each marker and subtracted from the apparent AUC to obtain the optimism‐corrected AUC. Because the markers were evaluated in the same small dataset, these analyses were considered exploratory and required validation in an independent cohort. Cutoff‐specific sensitivity and specificity values were not included in this study. Revised logistic regression and bootstrap ROC analyses were performed using Python version 3.14.4 with pandas version 2.3.3, NumPy version 2.4.4, statsmodels version 0.14.6, and scikit‐learn version 1.8.0. Other analyses were performed using SAS software (version 9.2; SAS Institute, Cary, NC, USA). Values of *p* < 0.05 were considered statistically significant.

**TABLE 1:** Patient characteristics and laboratory analyses.

	Blanket group (*n* = 28)	Control group (*n* = 27)	*p*
Male (%)	16 (57)	13 (48)	0.513
Age (years)	73.9 ± 8.5	75.8 ± 9.9	0.308
Body mass index (kg/m^2^)	24.5 ± 4.1	24.9 ± 4.3	0.820
CHADS_2_ score	2.71 ± 1.56	2.37 ± 1.28	0.404
*Laboratory analysis*			
AST (IU/L)	23.8 ± 5.4	21.9 ± 5.2	0.144
ALT (IU/L)	24.2 ± 9.9	19.9 ± 8.6	0.077
Hemoglobin (g/dL)	13.2 ± 1.7	12.8 ± 1.4	0.288
Serum creatinine (mg/dL)	0.82 ± 0.23	0.80 ± 0.27	0.602
Platelet count (×10^3^/μL)	206 ± 69	204 ± 64	0.729
PT (sec)	13.9 ± 3.9	13.0 ± 2.2	0.585
APTT (sec)	40.7 ± 11.7	38.9 ± 7.6	0.990
*Echocardiogram analysis*			
Left ventricular ejection fraction (%)	67.0 ± 7.7	67.0 ± 7.6	0.710
Left atrial diameter (mm)	36.7 ± 4.3	35.3 ± 6.8	0.284
*Medications on admission*			
Oral anticoagulation with NOAC	28 (100%)	27 (100%)	1.000
Apixaban 10 mg/day	16 (57%)	16 (59%)	0.876
Apixaban 5 mg/day	1 (4%)	1 (4%)	0.980
Edoxaban 60 mg/day	6 (21%)	7 (26%)	0.701
Edoxaban 30 mg/day	4 (14%)	1 (4%)	0.179
Rivaroxaban 15 mg/day	1 (4%)	1 (4%)	0.980
Rivaroxaban 10 mg/day	0 (0%)	0 (0%)	1.000
Dabigatran 300 mg/day	0 (0%)	1 (4%)	0.313
Dabigatran 220 mg/day	0 (0%)	0 (0%)	1.000
Vitamin K antagonist	0 (0%)	0 (0%)	1.000
Platelet inhibitor	0 (0%)	0 (0%)	1.000

*Note:* The numerical results are expressed as mean ± standard deviation.

Abbreviations: ALT, alanine transaminase; APTT, activated partial thromboplastin time; AST, aspartate transaminase; NOAC, non–vitamin K antagonist oral anticoagulant; PT, prothrombin time.

**TABLE 2:** Temperature evaluation, cryoablation procedures, and Modified Aldrete score evaluation.

	Blanket group (*n* = 28)	Control group (*n* = 27)	*p*
*Temperature evaluation*			
Esophageal hypothermia (%)	19 (68)	25 (93)	0.022
Skin hypothermia (%)	12 (43)	13 (48)	0.700
Pre‐esophageal temperature (°C)	36.2 ± 0.6	36.3 ± 0.6	0.767
Pre‐skin temperature (°C)	36.5 ± 0.3	36.5 ± 0.2	0.420
Post‐esophageal temperature (°C)	35.6 ± 0.6	34.9 ± 1.1	0.009
Post‐skin temperature (°C)	36.1 ± 0.4	35.9 ± 0.5	0.131
Δ in esophageal temperature (°C)	−0.7 ± 0.7	−1.4 ± 1.0	0.005
Δ in skin temperature (°C)	−0.5 ± 0.5	−0.6 ± 0.4	0.247
*Cryoablation procedures*			
Procedure time (min)	90 ± 23	90 ± 25	0.846
Number of cryoballoon applications	4.9 ± 0.8	5.1 ± 0.8	0.387
*Cooling time*			
Total (sec)	915 ± 196	928 ± 171	0.589
Right superior pulmonary vein (sec)	190 ± 44	203 ± 69	0.728
Right inferior pulmonary vein (sec)	221 ± 74	238 ± 78	0.383
Left superior pulmonary vein (sec)	304 ± 107	275 ± 90	0.100
Left inferior pulmonary vein (sec)	200 ± 48	212 ± 71	0.666
Touch‐up ablation	0 (0%)	1 (4%)	0.313
Cavo‐tricuspid isthmus ablation (%)	1 (4)	2 (7)	0.540
Nadir esophageal temperature (°C)	22.2 ± 5.2	21.7 ± 4.6	0.676
Nadir balloon temperature (°C)	−53.6 ± 4.0	−53.0 ± 3.6	0.535
Average bispectral index	48 ± 7	49 ± 5	0.933
Final activated clotting time before hemostasis (sec)	354 ± 19	349 ± 15	0.279
Protamine volume before hemostasis (mg/kg body weight)	0.96 ± 0.22	0.89 ± 0.26	0.422
Propofol volume (mg/kg body weight)	9.68 ± 2.97	9.33 ± 2.93	0.660
Dexmedetomidine volume (g/kg body weight)	1.05 ± 0.33	1.07 ± 0.39	0.906
Dopamine volume (mg/kg body weight)	0.47 ± 0.44	0.54 ± 0.43	0.324
Prevalence of dopamine use (%)	24 (86)	26 (96)	0.179
Time to hemostasis (min)	17.9 ± 6.7	19.2 ± 11.3	0.866
Complications (%)	0 (0)	1 (4)	0.313
Prevalence of wean off dopamine (%)	16 (57)	16 (59)	0.876
*Modified Aldrete score*			
Total	8.39 ± 0.99	7.70 ± 1.14	0.020
Motoric activity	1.96 ± 0.19	1.85 ± 0.36	0.153
Breathing	1.96 ± 0.19	1.93 ± 0.27	0.540
Blood pressure compared to reference measurement	1.68 ± 0.55	1.44 ± 0.58	0.129
Consciousness	1.36 ± 0.49	1.26 ± 0.45	0.442
Oxygen saturation	1.43 ± 0.50	1.22 ± 0.42	0.107
Prevalence of a Modified Aldrete score ≥ 9 (%)	18 (64)	10 (37)	0.044
Post‐anesthesia care unit stay duration (hours)	12.5 ± 6.8	12.8 ± 6.0	0.873

*Note:* The numerical results are expressed as mean ± standard deviation.

**TABLE 3:** Odds ratios for achievement of a Modified Aldrete score ≥ 9 at 2 h after discontinuation of anesthesia by randomized study group.

Study group	No. of patients	No. achieving MAS > = 9	Achievement of MAS > = 9, %	Odds ratio (95% CI)		
				Model 1	Model 2	Model 3
Control group (conventional towelettes)	27	10	37.0	1.00 (reference)	1.00 (reference)	1.00 (reference)
Heat insulation blanket group	28	18	64.3	3.06 (1.02–9.18)	3.02 (0.97–9.39)	3.15 (0.98–10.18)
*p*				0.046	0.056	0.055

*Note:* The outcome was achievement of a Modified Aldrete score >= 9 at 2 h after discontinuation of anesthesia. The Control group was the reference category. Odds ratios > 1 indicate higher odds of achieving MAS > = 9. Model 1 was unadjusted. Model 2 was adjusted for age (years) and left ventricular ejection fraction (LVEF; %). Model 3 was further adjusted for intra‐procedural cumulative propofol exposure (mg/kg) and dexmedetomidine exposure (microgram/kg). Models 2 and 3 were post hoc sensitivity analyses; covariates were selected on clinical grounds and not by univariable *p* values. *p* values are two‐sided Wald tests from the corresponding logistic regression models. Because anesthetic exposure was measured after randomization and may be on the causal pathway, Model 3 should not be interpreted as an estimate of the total effect of blanket assignment. All 55 participants had complete data for the displayed covariates.

Abbreviations: CI, confidence interval; LVEF, left ventricular ejection fraction; MAS, Modified Aldrete score; OR, odds ratio.

## 3 Results

### 3.1 Patient Characteristics and Laboratory Analysis (Table 1)

There were 28 and 27 patients in the Blanket and Control groups, respectively. No significant differences were observed in the proportion of male patients; age; body mass index; CHADS_2_ score; aspartate transaminase, alanine transaminase, hemoglobin, and serum creatinine levels; platelet counts; prothrombin time; activated partial thromboplastin time; or LVEF or LA diameter on echocardiography before cryoablation. All patients were administered oral anticoagulants with non–vitamin K oral anticoagulants (NOACs). No significant intergroup differences were observed in the types of NOACs administered. None of the patients were administered vitamin K antagonists or platelet inhibitors.

### 3.2 Temperature Evaluation (Table 2, Figure 4A–C)

**FIGURE 4:**
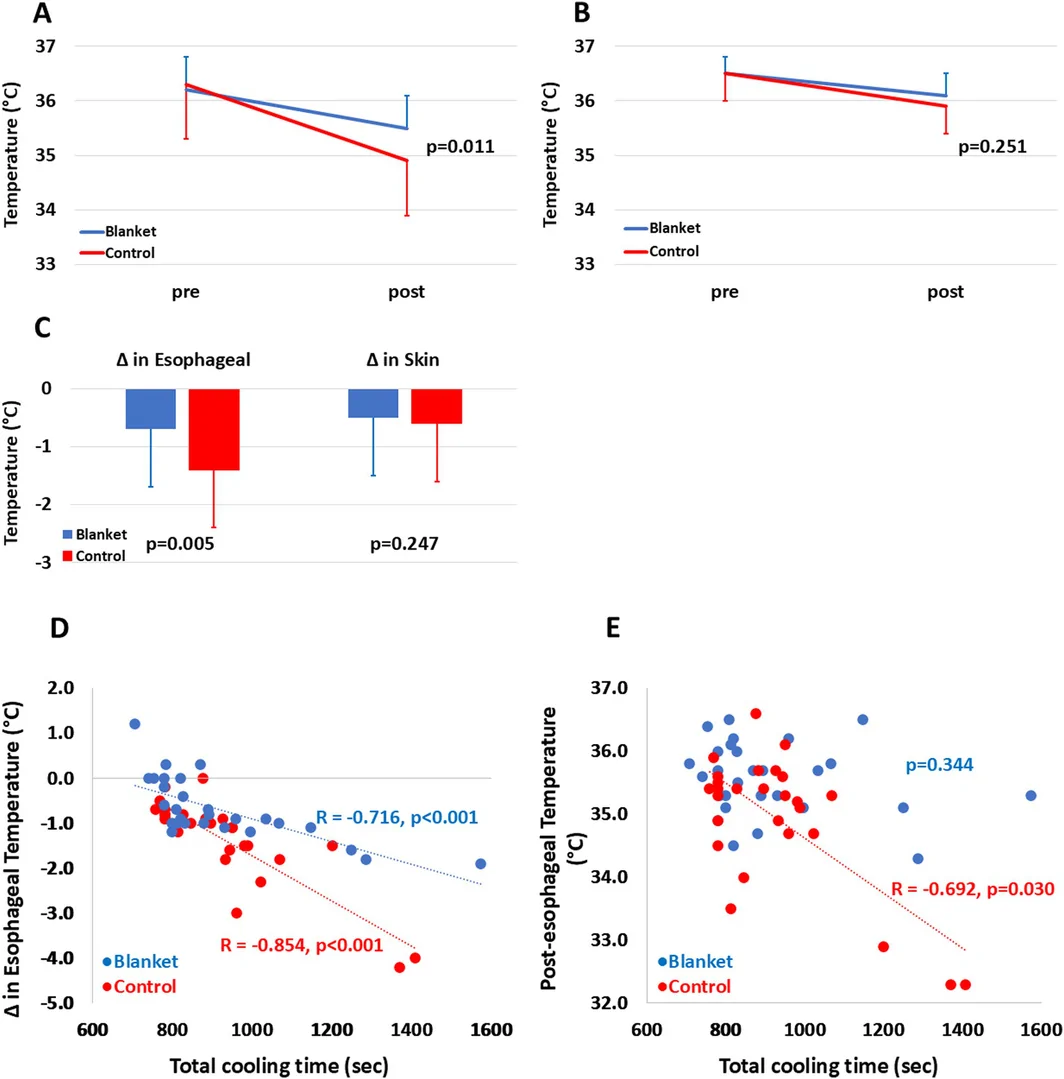
The esophageal (A) and skin (B) temperatures pre‐ and post‐cryoablation and Δ in the esophageal and skin temperatures (C) in the Blanket (blue lines and bars) versus Control (red lines and bars) groups. The *p* value of 0.005 shown for esophageal temperature represents the group‐by‐time interaction from the repeated‐measures analysis of variance. The numerical results are expressed as mean ± standard deviation. Scatter plots showing the relationship between the Δ in esophageal temperatures and total cooling time during cryoablation (D) in the Blanket (blue circles) and Control (red circles) groups, and the relationship between the post‐esophageal temperature and total cooling time during cryoablation (E) in the Blanket (blue circles) and Control (red circles) groups.

Of the 28 and 27 patients in the Blanket and Control groups, 19 (68%) and 25 (93%) as well as 12 (43%) and 13 (48%) had esophageal or skin hypothermia, respectively (Table 2). Temperature management with HIBs significantly prevents esophageal hypothermia (68% vs. 93%, respectively; *p* = 0.022) compared with control management. No significant intergroup differences were observed in the prevalence of skin hypothermia (Table 2) or the pre‐esophageal (Figure 4A), pre‐skin (Figure 4B), post‐skin (Figure 4B), or Δ in skin temperatures (Figure 4C). However, the post‐esophageal temperature was significantly lower than the pre‐esophageal temperature in the Control group (Table 2, Figure 4A). However, the post‐esophageal temperature was significantly higher in the Blanket group than in the Control group (Figure 4A). Moreover, the Δ in esophageal temperature was significantly lower in the Blanket group than in the Control group (Figure 4C).

### 3.3 Relationship Between Δ in Esophageal or Post‐Esophageal Temperatures and Total Cooling Time During Cryoablation (Figure 4D,E)

A significant correlation was found between the Δ in esophageal temperatures and total cooling time during cryoablation both in the Blanket (*R* = −0.716, *p* < 0.001) (blue circles in Figure 4D) and Control (*R* = −0.854, *p* < 0.001) (red circles in Figure 4D) groups, and between the post‐esophageal temperature and total cooling time during cryoablation both in the Control group (*R* = −0.692, *p* = 0.030) (red circles in Figure 4E), but not between the post‐esophageal temperature and total cooling time during cryoablation both in the Blanket group (*p* = 0.344) (blue circles in Figure 4E). These findings indicate that the decrease of the Δ in esophageal temperatures was likely to be dose‐dependent on cryo‐energy.

### 3.4 Cryoablation Procedures, Procedure‐Associated Complications, and MAS Evaluation (Table 2)

There were no statistically significant intergroup differences in procedure time; number of cryoballoon applications; cryoablation cooling time; prevalence of touch‐up and CTI ablation; nadir esophageal and balloon temperatures; average BIS index; final ACT; protamine volume administered before hemostasis; total volumes of propofol, dexmedetomidine, and dopamine; and weaning off of dopamine. The time to hemostasis and prevalence of dopamine use tended to be shorter and lower, respectively, in the Blanket group than in the Control group; however, the intergroup differences were not statistically significant. No patient experienced hypothermia‐associated shivering. One patient in the Control group developed a procedure‐associated complication of an atrio‐venous fistula in the right femoral vein. However, no statistically significant intergroup differences were observed in the prevalence of complications. On the other hand, a MAS (8.39 ± 0.99 vs. 7.70 ± 1.14; *p* = 0.020) and the prevalence of a MAS ≥ 9 (64% vs. 37%; *p* = 0.044) at 2 h after the discontinuation of sedation/anesthesia was significantly higher in the Blanket group than in the Control group. There were no statistically significant differences among the five components of MAS and stay duration in the PACU, because patients who underwent GA in the afternoon routinely stayed in the PACU overnight.

### 3.5 Treatment‐Group Comparison and Exploratory ROC Analyses (Table 3 and Figure 5)

**FIGURE 5:**
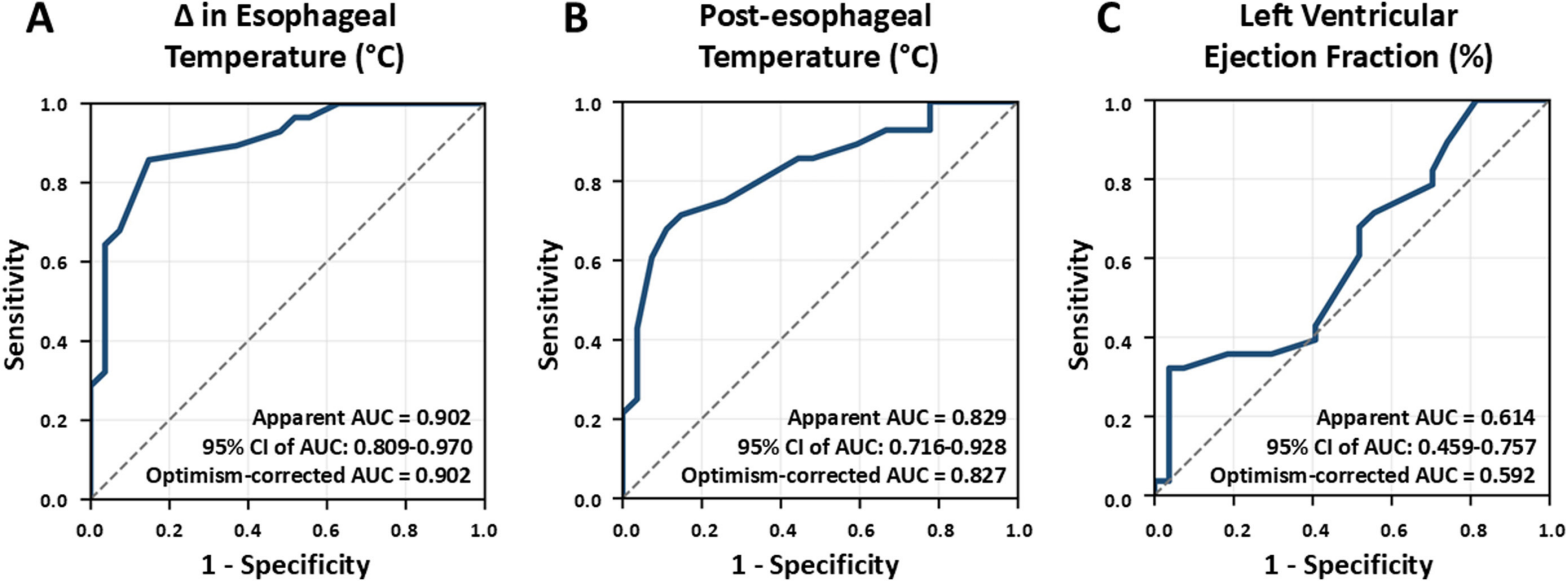
Exploratory receiver operating characteristic analyses of individual markers for achieving a Modified Aldrete score ≥ 9 at 2 h after discontinuation of sedation/anesthesia. Panels show (A) the Δ in esophageal temperature, calculated as post‐ minus pre‐esophageal temperature, with negative values indicating a decrease; (B) post‐esophageal temperature; and (C) left ventricular ejection fraction (LVEF). Apparent AUCs with 95% confidence intervals and bootstrap optimism‐corrected AUCs are shown. Internal assessment used 5000 outcome‐stratified bootstrap resamples. The markers were evaluated in the same small study dataset; therefore, these results are exploratory and require validation in an independent cohort. The *x*‐axis denotes 1–specificity. AUC, area under the curve; CI, confidence interval.

Among the 55 patients included in the analysis, 28 achieved MAS ≥ 9 at 2 h after discontinuation of anesthesia. MAS ≥ 9 was achieved by 18 of 28 patients (64%) in the Blanket group and 10 of 27 patients (37%) in the Control group. In Model 1 (Table 3), which included study group alone, HIB assignment was associated with higher odds of achieving MAS ≥ 9 (OR, 3.06; 95% CI, 1.02–9.18; *p* = 0.046). In the post hoc baseline‐adjusted sensitivity analysis including age and LVEF (Model 2), the magnitude of the association was similar, although the 95% CI included 1 (OR, 3.02; 95% CI, 0.97–9.39; *p* = 0.056). In Model 3, which additionally included cumulative propofol and dexmedetomidine exposure, the corresponding OR was 3.15 (95% CI, 0.98–10.18; *p* = 0.055). Model 3 was considered an exploratory conditional sensitivity analysis and was not interpreted as an estimate of the total effect of the blanket assignment.

The exploratory single‐marker ROC analyses are shown in Figure 5. For the Δ in esophageal temperature (Figure 5A), the apparent AUC was 0.902 (bootstrap 95% CI, 0.809–0.970) and the optimism‐corrected AUC was 0.902. For post‐esophageal temperature (Figure 5B), the apparent AUC was 0.829 (bootstrap 95% CI, 0.716–0.928) and the optimism‐corrected AUC was 0.827. For LVEF (Figure 5C), the apparent AUC was 0.614 (bootstrap 95% CI, 0.459–0.757) and the optimism‐corrected AUC was 0.592. These analyses were exploratory and did not aim to establish clinical decision thresholds.

## 4 Discussion

### 4.1 Main Findings in This Study

This study demonstrated that PTI using HIBs significantly reduced the incidence of esophageal hypothermia from 93% to 68% and increased the rate of successful early recovery from GA from 37% to 64% (Table 2).

### 4.2 Monitoring of Esophageal Temperatures

The esophagus site used in this study is reportedly the most suitable and accurate indicator of core body temperature because of its proximity to major blood vessels and the heart, and the monitoring values are highly reliable [1, 2, 3, 4]. Based on the findings shown in Figure 3, we considered that the esophageal temperature accurately reflected the core temperature 3 min after the final cryoballoon application. Previous reports demonstrated that GA usually decreases core temperature by 0.5°C–1.0°C [4]. In this study, since Δ in esophageal temperature was −1.4°C without PTI and/or passive warming, the cryoablation potentially exacerbated hypothermia under GA (Figure 4A). Moreover, the pre‐esophageal temperature was measured when the BIS was < 50, at which point the GA had already taken effect. Therefore, the influence of the pre‐esophageal temperature on the post‐esophageal temperature may have been underestimated.

### 4.3 Effect of Hypothermia on Time to Hemostasis

Although a recent report reported that perioperative hypothermia has negative effects on coagulation [1, 2, 3, 4], the time to hemostasis in the present study using Nepcell S in the Blanket group tended to be shorter than that in the Control group; however, no statistically significant intergroup difference was noted. We previously demonstrated that the use of the Nepcell S hemostatic pad was associated with shorter hemostasis time and fewer bleeding complications in patients undergoing cryoablation for AF [14]. Therefore, its use may accelerate the clotting cascade and shorten the time to hemostasis in patients with negative hypothermia‐induced effects on coagulation.

### 4.4 Cryoablation

Cryoablation [5, 6, 7, 8], with PVI as the index procedure for AF ablation, has favorable reported long‐term outcomes (8 years) in patients with AF [8], thereby identifying this procedure as a safe, effective, feasible, and useful treatment strategy for AF worldwide [5, 6, 7, 8] despite the current trend of reducing cryoablation volume due to pulsed‐field ablation [9].

### 4.5 Sedation/Anesthesia Modalities

Previous reports have demonstrated that patients are more prone to developing hypothermia when the operating room temperature is < 21°C [2, 4]. Therefore, we set the room temperature in the catheterization laboratory to 24°C during cryoablation. Sedation/anesthesia modalities may also play an important role in patient‐reported outcomes and resumption of activity. Previous [15] and recent [16] studies demonstrated that moderate sedation offered an alternative to GA that was safe and well‐tolerated by patients with comparable success rates and improved electrophysiological laboratory throughput [15]; moreover, conscious sedation is as safe and effective as GA, particularly with cryoablation [16], among patients who undergo ablation for AF.

However, recent studies have reported that GA reduces perceived pain, and patients administered GA are more motivated to repeat the procedure than those administered conscious sedation [17], leading to higher quality of life scores [18] among those who undergo cryoablation for AF. In fact, the Heart Rhythm Society Expert Consensus Statement task force members reported that GA is used in 73% of patients undergoing AF ablation [19]. Moreover, patients undergoing AF ablation have a high prevalence (89%) of sleep‐disordered breathing [20], which is associated with several major pathophysiological abnormalities, including intrathoracic pressure swings [21]. These swings in intrathoracic pressure during AF ablation may cause negative pressure in the cryosheath, which may be a risk factor for serious air embolism [22].

Our anesthesia strategy for GA with a supraglottic airway while monitoring BIS using HIBs may help maintain a stable respiratory condition [13], thus avoiding intrathoracic pressure swings and preventing hypothermia. Therefore, although the choice of sedation/anesthesia appears to be driven by patient characteristics and institutional factors without affecting complications [16], these findings suggest feasible and safe strategies for cryoablation for AF.

### 4.6 Potential Mechanisms of Prolonged Recovery From GA During Cryoablation

Hypothermia during GA develops due to three characteristic factors: (1) initial redistribution of body heat from the core to the periphery, (2) GA‐induced impairment of thermoregulatory control, and (3) heat loss exceeding metabolic heat production [1, 2, 3, 4]. The incidence of perioperative hypothermia was reportedly 4%–90% in some studies [1, 2, 3, 4]. The prevalence of esophageal hypothermia in the Control group (93%) of the present study might have been higher than in those reports [1, 2, 3, 4]. Thus, the placement of cryoballoons in the LA may more effectively exacerbate the decrease in esophageal temperature (Figure 3). Subsequently, cooled blood in the LA may be sent to the entire body, which may induce hypothermia. The correlation between Δ in esophageal temperature and total cooling time during cryoablation was statistically significant (Figure 4D).

Hypothermia decreases the metabolism of anesthesia, decreases blood flow to the liver and kidneys, and contributes to prolonged recovery time from GA. A recent report demonstrated that cryoablation affects the intrinsic cardiac nervous system, an epicardial neural system composed of ganglionated plexi and a complex network of interconnecting neurons [23, 24]. Neuromodulation during cryoablation increases vagal tone, leading to vasodilation, hypotension, and bradycardia, thereby decreasing the cardiac output [23, 24]. To achieve normothermia, the brain must obtain sufficient blood flow to wash out anesthesia and restore normal thermoregulation [1]. However, residual anesthesia in the brain caused by insufficient blood flow following increased vagal tone during cryoablation may weaken these responses.

### 4.7 Other Device Therapies

Although several intraoperative warming devices are commercially available [25], future studies are required to identify the most effective device.

### 4.8 Interpretation of Multivariate Analysis

The proportion of patients achieving MAS ≥ 9 at 2 h was higher in the Blanket group than in the Control group (64% vs. 37%; *p* = 0.044). The estimated OR for blanket assignment remained at approximately 3 across the unadjusted and adjusted models. However, the 95% CIs were wide and included 1 in Models 2 and 3. Therefore, the revised analyses support a possible association between HIB use and adequate early recovery, but they do not establish a definitive independent or causal effect. Therefore, further studies are required to obtain more accurate estimates. Post‐esophageal temperature, △ in esophageal temperature, and the hypothermia indicator were not included in the treatment‐group comparison models because these closely related post‐randomization measures may lie on the pathway between blanket assignment and recovery. Their separate ROC analyses should be interpreted as exploratory descriptions of marker discrimination, rather than as evidence that they are independent predictors.

### 4.9 Elderly Patient Characteristics

The mean age of our patient population (74.8 ± 9.1 years) [26] may be comparatively older than those of hospitals in other cities due to our hospital's location in Kitakyushu City, the oldest ordinance‐designated city in Japan (Table 1). Previous reports have demonstrated that elderly patients are more prone to hypothermia during GA because aging impairs autonomic thermoregulation and reduces muscle mass and metabolic heat production. Anesthetic drugs further depress thermoregulatory responses and cause peripheral vasodilation, which promotes heat loss and redistribution of core heat to the cooler extremities [1, 2, 3, 4, 27].

### 4.10 Study Limitations

This prospective study was limited by its single‐center design and relatively small number of patients. The mean patient age was higher than that reported in previous studies [5, 6, 7, 8]. Thus, in this study, older age may have been associated with a higher prevalence of hypothermia and prolonged recovery from GA [1, 27], and the effects of HIBs may have been limited. Moreover, other core temperature measurements (e.g., rectal and bladder) were not performed to validate the systemic nature of hypothermia, which might be a major limitation. However, the statistical analyses have additional limitations. The sample included only 55 patients, of whom 28 achieved MAS ≥ 9, resulting in limited precision for multivariable estimates and a risk of overfitting. Models 2 and 3 were post hoc sensitivity analyses rather than prespecified confirmatory models. Model 3 included post‐randomization anesthetic exposure and, therefore, represents a conditional analysis rather than the total effect of blanket assignment. Additionally, the three ROC markers were evaluated using the same dataset in which they were identified. Although bootstrap analysis was used to quantify uncertainty and estimate optimism‐corrected AUCs, it provides only an internal assessment and cannot replace external validation. Therefore, the ROC findings should be considered exploratory and hypothesis‐generating, and no clinical cutoff values can be established from the present study. Therefore, the generalizability of these results to multicenter trials that include other core temperature measurements and a larger number of patients with a younger mean age should be determined in future randomized clinical trials.

## 5 Conclusions

During cryoablation, 93% of patients treated without PTI experienced hypothermia, which may have prolonged recovery from GA. PTI using HIBs was associated with a smaller decline in esophageal temperature and a higher proportion of patients achieving MAS ≥ 9 at 2 h after discontinuation of anesthesia during cryoablation under GA. The adjusted estimates were similar in magnitude, but imprecise, and the exploratory ROC findings required external validation. Larger studies using validated core temperature measurements and clinically meaningful recovery endpoints are required.

## Author Contributions

All authors were responsible for the patients and study. Each ablation was performed by Dr. M. Takemoto, while the electrophysiological studies were performed in a cardiac catheterization laboratory by Dr. T. Tsuchihashi, R. Goya, Y. Sadasue, K. Nagaoka, N. Terauchi, H. Inoue, and M. Kurachi, managed the patients and delivered the passive warming treatment in the cardiac catheterization laboratory. Drs. T. Mito and T. Nagata, performed the statistical analyses. Ms. A. Yamaguchi, Dr. M. Takemoto, Ms. K. Matsuno, and Dr. T. Okamoto wrote the manuscript.

## Funding

The authors have nothing to report.

## Ethics Statement

This was a single‐center prospective clinical trial, and the procedures were performed in accordance with the Declaration of Helsinki and the ethical standards of the committee responsible for human experimentation. This study was approved by the Ethical Review Board of Social Medical Corporation Steel Memorial Yawata Hospital on October 24, 2022, under reference number 22‐58. This study was retrospectively registered in UMIN‐CTR under registration number UMIN000057904 on May 19, 2025. The study was originally initiated as a prospective nursing research project, and registration was delayed because full‐length journal publication was not initially planned. The study protocol, including the endpoints and statistical analysis plan, had been finalized before enrollment of the first patient, and no changes were made before registration.

## Consent

Written informed consent was obtained from all the patients.

## Conflicts of Interest

The authors declare no conflicts of interest.

## Supporting information


**Appendix S1:** Modified Aldrete score.

## Data Availability

Owing to the ethical restrictions imposed by the ethics committee and to protect patient privacy, raw data cannot be shared publicly.

## References

[1] D. I. Sessler , “Perioperative Heat Balance,” Anesthesiology 92 (2000): 578–596, 10.1097/00000542-200002000-00042. 10691247

[2] J. Yi , Y. Lei , S. Xu , et al., “Intraoperative Hypothermia and Its Clinical Outcomes in Patients Undergoing General Anesthesia: National Study in China,” PLoS One 12 (2017): e0177221, 10.1371/journal.pone.0177221. PMC5464536 28594825

[3] S. Rauch , C. Miller , A. Bräuer , B. Wallner , M. Bock , and P. Paal , “Perioperative Hypothermia‐A Narrative Review,” International Journal of Environmental Research and Public Health 18 (2021): 8749, 10.3390/ijerph18168749.

[4] N. Ji , J. Wang , X. Li , and Y. Shang , “Strategies for Perioperative Hypothermia Management: Advances in Warming Techniques and Clinical Implications: A Narrative Review,” BMC Surgery 24 (2024): 425, 10.1186/s12893-024-02729-0. PMC11684272 39736577

[5] A. Luik , A. Radzewitz , M. Kieser , et al., “Cryoballoon Versus Open Irrigated Radiofrequency Ablation in Patients With Paroxysmal Atrial Fibrillation: The Prospective, Randomized, Controlled, Noninferiority FreezeAF Study,” Circulation 132 (2015): 1311–1319, 10.1161/CIRCULATIONAHA.115.016871. PMC4590523 26283655

[6] J. G. Andrade , G. A. Wells , M. W. Deyell , et al., “Cryoablation or Drug Therapy for Initial Treatment of Atrial Fibrillation,” New England Journal of Medicine 384 (2021): 305–315, 10.1056/NEJMoa2029980. 33197159

[7] O. M. Wazni , G. Dandamudi , N. Sood , et al., “Cryoballoon Ablation as Initial Therapy for Atrial Fibrillation,” New England Journal of Medicine 384 (2021): 316–324, 10.1056/NEJMoa2029554. 33197158

[8] M. Schiavone , G. Molon , P. Pieragnoli , et al., “Very Long‐Term Follow‐Up of Pulmonary Vein Isolation Using Cryoballoon for Catheter Ablation for Atrial Fibrillation: An 8‐Year Multicenter Experience,” Circulation. Arrhythmia and Electrophysiology 18 (2025): e013645, 10.1161/CIRCEP.124.013645. 40709468

[9] T. Reichlin , T. Kueffer , P. Badertscher , et al., “Pulsed Field or Cryoballoon Ablation for Paroxysmal Atrial Fibrillation,” New England Journal of Medicine 392 (2025): 1497–1507, 10.1056/NEJMoa2502280. 40162734

[10] J. A. Aldrete , “The Post‐Anesthesia Recovery Score Revisited,” Journal of Clinical Anesthesia 7 (1995): 89–91, 10.1016/0952-8180(94)00001-K. 7772368

[11] K. Ono , Y. K. Iwasaki , M. Akao , et al., “JCS/JHRS 2020 Guideline on Pharmacotherapy of Cardiac Arrhythmias,” Circulation Journal 86 (2022): 1790–1924, 10.1253/circj.CJ-20-1212. 35283400

[12] T. Nakano , T. Oka , K. Okayama , et al., “Association Between Oral Anticoagulants Continuation on Thromboembolism and Bleeding Events in Patients With CHADS(2),” Journal of Cardiology 86 (2025): 359–365, 10.1016/j.jjcc.2025.04.004. 40287088

[13] S. Hida , M. Takemoto , A. Masumoto , et al., “Clinical Benefits of Deep Sedation With a Supraglottic Airway While Monitoring the Bispectral Index During Catheter Ablation of Atrial Fibrillation,” Journal of Arrhythmia 33 (2017): 283–288, 10.1016/j.joa.2017.04.001. PMC5529590 28765758

[14] R. Goya , M. Takemoto , E. Nyuta , et al., “Efficacy and Safety of Nepcell S in Achieving Hemostasis After Removal of a 15‐Fr Femoral Venous Sheath in Patients Undergoing Cryoballoon Ablation for Atrial Fibrillation,” Circulation Reports 3 (2021): 691–698, 10.1253/circrep.CR-21-0105. PMC8651474 34950794

[15] J. Wasserlauf , R. M. Kaplan , D. R. Walega , et al., “Patient‐Reported Outcomes After Cryoballoon Ablation Are Equivalent Between Moderate Sedation and General Anesthesia,” Journal of Cardiovascular Electrophysiology 31 (2020): 1579–1584, 10.1111/jce.14547. PMC9451135 32400079

[16] E. Massalha , A. Dakka , A. Sabbag , et al., “Comparative Analysis of Anaesthesia Modalities in Pulmonary Vein Isolation: Insights From a Prospective Multicentre Registry,” Europace 27 (2025): euae301, 10.1093/europace/euae301. PMC11831030 39957475

[17] M. Nesti , F. Lucà , G. Mirizzi , et al., “Patient‐Reported Outcomes After First Pulmonary Vein Isolation for ParoxYsmal Atrial Fibrillation: Cryoballoon vs. Radiofrequency (SPY‐AF),” Journal of Clinical Medicine 14 (2025): 6711, 10.3390/jcm14196711. PMC12525301 41095791

[18] A. Matteucci , M. Russo , M. Galeazzi , et al., “Impact of Ablation Energy Sources on Perceived Quality of Life and Symptom in Atrial Fibrillation Patients: A Comparative Study,” Journal of Clinical Medicine 14 (2025): 2741, 10.3390/jcm14082741. PMC12028017 40283571

[19] H. Calkins , G. Hindricks , R. Cappato , et al., “2017 HRS/EHRA/ECAS/APHRS/SOLAECE Expert Consensus Statement on Catheter and Surgical Ablation of Atrial Fibrillation,” Heart Rhythm 14 (2017): e275–e444, 10.1016/j.hrthm.2017.05.012. PMC6019327 28506916

[20] N. Tanaka , K. Tanaka , Y. Hirao , et al., “Home Sleep Apnea Test to Screen Patients With Atrial Fibrillation for Sleep Apnea Prior to Catheter Ablation,” Circulation Journal 85 (2021): 252–260, 10.1253/circj.CJ-20-0782. 33298643

[21] M. R. Cowie , D. Linz , S. Redline , V. K. Somers , and A. K. Simonds , “Sleep Disordered Breathing and Cardiovascular Disease: JACC State‐of‐the‐Art Review,” Journal of the American College of Cardiology 78 (2021): 608–624, 10.1016/j.jacc.2021.05.048. 34353537

[22] K. Tsukahara , Y. Oginosawa , Y. Fujino , et al., “Prevention of Serious Air Embolism During Cryoballoon Ablation; Risk Assessment of Air Intrusion Into the Sheath by Catheter Selection and Change in Intrathoracic Pressure: An Ex Vivo Study,” Journal of Cardiovascular Electrophysiology 30 (2019): 2944–2949, 10.1111/jce.14208. 31588621

[23] A. Del Monte , L. Pannone , A. Bisignani , et al., “Cryoballoon Ablation for Atrial Fibrillation: Effects on Neuromodulation,” Frontiers in Cardiovascular Medicine 9 (2022): 958316, 10.3389/fcvm.2022.958316. PMC9366392 35966567

[24] N. H. Waldron , M. Fudim , J. P. Mathew , and J. P. Piccini , “Neuromodulation for the Treatment of Heart Rhythm Disorders,” JACC: Basic to Translational Science 4 (2019): 546–562, 10.1016/j.jacbts.2019.02.009. PMC6712352 31468010

[25] H. Xu , Z. Wang , Y. Lu , et al., “Value of Active Warming Devices for Intraoperative Hypothermia Prevention‐A Meta‐Analysis and Cost–Benefit Analysis,” International Journal of Environmental Research and Public Health 18 (2021): 11360, 10.3390/ijerph182111360. PMC8582721 34769877

[26] C. Xue , “Extensive Ablation in Elderly Patients With Persistent AF: Insights and Considerations,” Journal of Cardiology 86 (2025): 318, 10.1016/j.jjcc.2025.05.006. 40436137

[27] S. Han , W. Ou , D. Zheng , L. Chen , and X. Lin , “Characteristics of Intraoperative Hypothermia and Rewarming Responses in Elderly Patients Undergoing General Anesthesia Across Different Traditional Chinese Medicine Constitutions,” Journal of Perianesthesia Nursing (2026): S1089‐9472(26)00144–9, 10.1016/j.jopan.2026.05.004. 42446443

