# Supplementary material for: Association Between Temperature Management Using Passive Thermal Insulation and a Modified Aldrete Score During Cryoablation of Paroxysmal Atrial Fibrillation Under General Anesthesia
Source: J Arrhythm. 2026 Sep 24;42(5):e70460. doi: 10.1002/joa3.70460 (PMC13602821; doi:10.1002/joa3.70460)
Supplement: Supplementary file 1 [file JOA3-42-e70460-s001.docx]

**Supplementary material**

**Modified Aldrete score**^1^

| **Motoric activity** |  |
| --- | --- |
| ● Spontaneous movement when addressed | **2** |
| ● Weak spontaneous movement when addressed | **1** |
| ● No movement | **0** |
| **Breathing** |  |
| ● Coughs on comment or cries | **2** |
| ● Keeps the airway open | **1** |
| ● Obstructed airways | **0** |
| **Blood pressure compared to reference measurement*** |  |
| ● Δ < 20 mmHg | **2** |
| ● Δ = 20 - 50 mmHg | **1** |
| ● Δ > 50 mmHg | **0** |
| **Consciousness** |  |
| ● Awake | **2** |
| ● Response to stimulus, reflexes intact | **1** |
| ● No answer, reflexes absent | **0** |
| **Oxygen saturation** |  |
| ● 100 - 98% | **2** |
| ● 97 – 95% | **1** |
| ● < 95% | **0** |
| *****Reference measurement was performed 11/2 minutes after |  |
| administration of the spasmolytic agent. |  |

1. Aldrete JA. The post-anesthesia recovery score revisited. J Clin Anesth. 1995;7:89-91.
